# The Tobacco Habit

**Published:** 1892-05

**Authors:** 


### THE TOBACCO HABIT.

Statistics as to the effects of tobacco smoking upon students have been collected at Amherst and Yale colleges. The non-smokers at Amherst are of greater weight than the smokers; they are superior in chest girth to the smokers, and their lung capacity is higher. The non-smokers are more athletic than the smokers, and more successful in athletic sports. The non-smokers at Amherst, as at Yale, have also an advantage over the smokers in mental power and in scholarship. The facts recently collected in American colleges concerning the physiological and physical effects of the tobacco smoking habit are instructive to the young men who go to college, and also to those who do not.

